# EEG Motor Imagery Classification: Tangent Space with Gate-Generated Weight Classifier

**DOI:** 10.3390/biomimetics9080459

**Published:** 2024-07-27

**Authors:** Sara Omari, Adil Omari, Fares Abu-Dakka, Mohamed Abderrahim

**Affiliations:** 1 Department of System Engineering and Automation, University Carlos III of Madrid, Avda de la Universidad 30, 28911 Leganes, Spain; somari@ing.uc3m.es; 2 Department of Signal Theory and Communications, University Carlos III of Madrid, Avda de la Universidad 30, 28911 Leganes, Spain; aomari@ing.uc3m.es; 3 Electronic and Computer Science Department, Faculty of Engineering, Mondragon Unibertsitatea, 20500 Arrasate, Spain; fabudakka@mondragon.edu

**Keywords:** brain–computer interface, motor imagery, tangent space, GG-FWC, classification, gender-based analysis

## Abstract

Individuals grappling with severe central nervous system injuries often face significant challenges related to sensorimotor function and communication abilities. In response, brain–computer interface (BCI) technology has emerged as a promising solution by offering innovative interaction methods and intelligent rehabilitation training. By leveraging electroencephalographic (EEG) signals, BCIs unlock intriguing possibilities in patient care and neurological rehabilitation. Recent research has utilized covariance matrices as signal descriptors. In this study, we introduce two methodologies for covariance matrix analysis: multiple tangent space projections (M-TSPs) and Cholesky decomposition. Both approaches incorporate a classifier that integrates linear and nonlinear features, resulting in a significant enhancement in classification accuracy, as evidenced by meticulous experimental evaluations. The M-TSP method demonstrates superior performance with an average accuracy improvement of 6.79% over Cholesky decomposition. Additionally, a gender-based analysis reveals a preference for men in the obtained results, with an average improvement of 9.16% over women. These findings underscore the potential of our methodologies to improve BCI performance and highlight gender-specific performance differences to be examined further in our future studies.

## 1. Introduction

Brain–computer interfaces (BCIs) have emerged as a revolutionary technology in the field of neuroscience and biomedical engineering. These interfaces establish a direct communication channel between the brain and external devices or computer systems, bypassing conventional neuromuscular pathways. By deciphering neural activity patterns, a brain–computer interface (BCI) enables individuals to interact with their environment and control assistive devices, and it even restores lost sensory or motor functions [1]. Among the diverse BCI paradigms, motor imagery brain–computer interfaces (MI-BCIs) have become a prominent approach: leveraging the brain’s ability to generate neural signals during imagined motor actions [2]. In MI-BCI systems, electroencephalogram (EEG) serves as a primary modality for capturing neural activity due to its non-invasive nature, high temporal resolution, and portability [3].

In the realm of MI-BCIs, the accurate classification of imagined motor actions is crucial for effective system performance [4]. Covariance matrices have become a fundamental tool in this context due to their ability to capture the statistical dependencies between EEG channels during motor imagery (MI) tasks. These matrices provide a compact and informative representation of the spatial patterns of brain activity during MI tasks. By modeling the covariance structure of EEG signals, researchers can extract features that reflect the underlying neural processes associated with different MI states. Recent studies have explored various feature extraction and classification techniques to enhance the performance of MI-BCIs. For instance, Al-Qazzaz et al. introduced a robust framework for EEG feature fusion aimed at enhancing MI-BCIs for stroke patients [5] and discussed the use of signal complexity measurements to improve BCI systems for stroke rehabilitation [6].

Covariance matrices are often utilized within the framework of Riemannian geometry, which allows for the effective analysis and classification of these matrices. The use of Riemannian geometry enables the computation of distances and means in a manner that respects the intrinsic geometric properties of the covariance matrices. This approach has been shown to enhance the discrimination between different motor imagery tasks, leading to improved classification accuracy in MI-BCI systems [7].

This paper aims to analyze and classify covariance matrices, with our vision extending towards future applications in the medical field of robotics and control. Specifically, we envision the utilization of our methodology for controlling prosthetics, exoskeletons, and robotic arms in order to improve the quality of life of individuals with motor impairments. This interdisciplinary approach is promising for advancing the field of medicinal robotics, with the potential to revolutionize rehabilitation practices and assistive technologies.

Despite the advancements in utilizing covariance matrices and Riemannian geometry for MI-BCIs, several gaps remain. One significant challenge is the variability of EEG signals over time and across individuals. To address this issue, we propose using covariance matrices combined with multiple tangent space projection (M-TSP). M-TSP involves projecting covariance matrices into multiple tangent spaces, with each tangent space providing the classifier with a different perspective of the data depending on the class. This method captures and refines statistical dependencies between EEG channels, providing a robust framework that enables traditional machine learning algorithms to more effectively manage such variability.

The resulting outputs from these methodologies serve as inputs to the gate-generated functional weight classifier (GG-FWC) classifier, which constructs the output through a functionally weighted linear combination of the input variables. The GG-FWC classifier improves classification accuracy while avoiding the extensive data and computational demands typically associated with deep learning techniques. This choice is motivated by the good results obtained using linear classifiers [8] and because GG-FWC can be interpreted as a weighted sum of the original features and features resulting from nonlinear transformations based on the training dataset topology [8]. This approach allows for the detection of different types of patterns in the data, suggesting a potential significant improvement.

Another challenge is the presence of noise from muscle movements, eye blinks, and electronic interference. Our proposed method mitigates noise from muscle movements, eye blinks, and electronic interference by focusing on channel relationships through covariance matrices. This enhances signal robustness by allowing the GG-FWC classifier to effectively distinguish relevant patterns from noise. These innovations collectively enhance the robustness, accuracy, and efficiency of motor imagery BCI systems, contributing to more effective signal processing and classification.

Moreover, there is a lack of comprehensive studies comparing different covariance matrix analysis methodologies such as tangent space projections and Cholesky decomposition. Our study performs a comparative analysis between these two approaches, highlighting their respective advantages and limitations. Furthermore, most studies do not address potential gender differences in MI-BCI performance, which could provide insights into personalized BCI system design. We conduct a gender-based performance analysis to investigate potential differences in MI-BCI accuracy between male and female subjects, providing insights for more personalized and effective BCI designs.

By addressing the inherent challenges of EEG signal variability and noise, our model contributes to more reliable and user-friendly BCI applications. This approach holds significant promise for advancing medicinal robotics and assistive technologies, potentially revolutionizing rehabilitation practices for individuals with motor impairments. In summary, this paper aims to address these gaps through the following contributions:

– Introduction of the GG-FWC classifier for categorizing covariance matrices extracted from EEG signals;
– Integration of both linear and nonlinear features to enhance classification accuracy;
– Comprehensive comparison of M-TSP and Cholesky decomposition methodologies;
– Mitigation of noise from muscle movements, eye blinks, and electronic interference by focusing on channel relationships through covariance matrices;
– Gender-based performance analysis to investigate potential differences in MI-BCI accuracy between male and female subjects.

The remainder of the paper is organized as follows. The state-of-the-art is presented in the next section: Section 2. The proposed method is presented in Section 3 and is validated using datasets 2a and 2b from the BCI Competition IV, as introduced in Section 4. The results and corresponding discussions are presented Section 4.2. Additionally, the manuscript includes a gender-based examination in Section 4.3, contributing further insights to the analysis. Finally, Section 5 and Section 6 provide a discussion, future research directions, and conclusions of this study.

## 2. Related Works

In the realm of classifying EEG signals, two primary strategies emerge: feature engineering and deep learning methodologies. Feature engineering involves extracting pertinent features from the EEG signal, which are then utilized as inputs for conventional classifiers [9]. On the other hand, a more advanced approach harnesses deep learning methodologies: specifically, convolutional neural networks (CNNs) and long short-term memory (LSTM) networks [10]. These techniques possess the inherent capability to directly process the unprocessed raw EEG signals, bypassing the need for explicit feature extraction.

In the last decade, the utilization of covariance matrices and Riemannian geometry for processing EEG signals has experienced significant growth. This approach, originally introduced by Barachant et al. [11], uses spatial covariance matrices as EEG signal descriptors and employs Riemannian geometry to directly classify these matrices using the topology of the manifold of symmetric positive definite (SPD) matrices. Within this context, two methodologies have been proposed. The first [11] involves the classification of covariance matrices directly within their native Riemannian manifold. In this case, the classifier filter geodesic minimum distance to Riemannian mean (FGMDRM) computes the Riemannian mean of the covariance matrices for each class and assigns incoming samples to the class with the nearest mean for classification. This method has been improved by Singh et al. [12], who introduced spatial filtering of EEG signals regularized by data from other subjects. This method enables the reduction in the dimensionality of covariance matrices, which, in turn, reduces the number of samples needed to train the model and adapt it to a new user.. The advantage of this approach lies in the treatment of SPD matrices within their native space, thereby ensuring the preservation of information. However, the need to adapt existing classifiers or develop new ones to suit this framework poses a limitation.

To address this issue, the second approach [11] consists of mapping the covariance matrices from their native manifold space to a tangent space, which is a Euclidean space. In tangent space, matrices can be vectorized and treated as Euclidean objects, making them amenable to classification using standard algorithms. Barachant et al. in [13] presented the tangent space linear discriminant analysis (TSLDA) algorithm, which begins by projecting covariance matrices from their native manifold into a tangent space at the training set covariance matrices’ Riemannian mean. Later, linear discriminant analysis (LDA) is employed for sample classification. In our previous work [8], we introduced a multiple tangent space projection technique whereby the entire dataset is projected into tangent spaces, with each space corresponding to the Riemannian mean of a specific class. Subsequently, the resulting data representations are concatenated, and the logistic regression (LR) and support vector machine (SVM) classifiers are utilized for classification. Unlike [8], in this paper, we explore the potential of a nonlinear classifier with a significantly greater expressive power: namely, the GG-FWC, which can be viewed as a linear model wherein the constant weights have been replaced with functions of the model inputs.

To mitigate the non-stationarity of EEG signals, Gaur et al. [14] suggested utilizing subject-specific multivariate empirical mode decomposition (SS-MEMD) to identify the optimal frequency band for the particular MI task. This process results in an enhanced EEG signal, from which covariance matrices are derived and then classified using LR within the tangent space framework. To address the high dimensionality of the Riemannian manifold, Xie et al. [15] proposed a bilinear mapping strategy that transforms the native covariance matrix manifold into a lower-dimensional manifold while preserving the data distribution. This methodology mitigates overfitting and reduces the computational load. Following this transformation, a classifier can be applied either within the manifold itself or in the tangent space.

Several models have been proposed that, similar to our approach, make use of multiple projections onto different tangent spaces. For example, Miah et al. [16] introduced a multi-tangent space projection method whereby the tangent spaces are dependent on subbands of the EEG signal. Specifically, they proposed dividing the EEG signal into five subbands and projecting the sample covariance matrix of each subband into the respective tangent space. Afterward, principal component analysis (PCA) is employed to reduce the dimensionality of the resulting features, followed by classification using SVM. In [17], Fang et al. aggregated an initial step to the aforementioned proposal that segments the EEG signal into distinct time windows. These methods improve the performance compared to using a single projection but encounter the problem of generating a high-dimensional feature space, which requires adding a dimensionality reduction step or using a classifier resistant to this curse to prevent overfitting. All of these models focused on generating projections onto tangent spaces that depend solely on the EEG signal’s time and frequency division (frequency subbands and time windowing). In contrast, our work focuses on generating class-dependent projections.

An interesting study to be considered in future work was presented in [18], which explores alternative descriptors for covariance matrices other than tangent space in the baseline analysis. In particular, four alternatives are considered: eigenvalues, eigenvectors, coefficients of the characteristic polynomial (where roots represent eigenvalues), and the spectral radius (the maximum eigenvalue).

Besides BCI, the application of Riemannian geometry to work with SPD matrices has gained increasing prominence in machine learning tasks across various fields, such as learning-from-demonstration [19], medical imaging [20], and visual object categorization [21].

## 3. Methodology

### 3.1. Multiple Tangent Space Projections

The tangent space classification (TSC) method offers an innovative approach to deal with the complexities associated with using computationally intricate Riemannian geometry and developing classifiers based on that geometry. Instead, this method optimizes a more direct route by projecting the data into an Euclidean space. In this Euclidean space, the application of standard classifiers becomes more accessible and efficient, providing a practical alternative to navigating the complexities of Riemannian geometry. This approach is expressed succinctly in the work of Barachant [7].

The implementation of the TSC is developed through four sequential steps:

– Computation of covariance matrices: The covariance matrices of EEG signal trials are computed. Each covariance matrix represents the statistical dependencies between different EEG channels.
– Definition of tangent space: A reference point on the manifold, typically the Riemannian mean of the training set covariance matrices, is chosen. This point defines the tangent space.
– Projection onto tangent space: The covariance matrices are projected onto the tangent space using the logarithm map. This projection transforms the covariance matrices from the Riemannian manifold to a Euclidean space.
– Application of standard classifiers: In the Euclidean space of the tangent space, standard classifiers such as logistic regression or support vector machines are applied.

Multiple tangent space classification (M-TSC), presented in [8], involves the utilization of multiple tangent space projections, each corresponding to a distinct class, affording the classifier distinct perspectives of the data based on the respective class. This strategy is driven by the objective of generating class-specific and discriminatively enriched features, thus contributing to the establishment of a robust and precise model for pattern recognition and prediction.

M-TSC is characterized by the projection of input data into distinct tangent spaces utilizing designated reference points $C_{ref0}$, $C_{ref1},\ldots ,C_{refM}$, which represent the Riemannian means of training samples related to classes m=1,2,…,M. The resulting projection matrices, denoted as $Z_{0}^{\left(n\right)}$, $Z_{1}^{\left(n\right)},\ldots ,Z_{M}^{\left(n\right)}$, are calculated as follows:

(1) $$Z_{m}^{\left(n\right)}=C_{refm}^{1/2}\log\left(C_{refm}^{-1/2}C^{\left(n\right)}C_{refm}^{-1/2}\right)C_{refm}^{1/2}$$

where n=1,…,N represents the index of the samples, with *N* being the number of samples.

Given the symmetry inherent in $Z_{m}^{\left(n\right)}$ and recognizing the unsuitability of redundant input features for machine learning algorithms, consideration is given to the upper triangular segment of the matrix. This selected portion is subsequently rearranged to form a vector of length d=e(e+1)/2, where *e* denotes the number of EEG channels. The reformulation of (1) is thus articulated as:

(2) $$z_{m}^{\left(n\right)}=upper\left(C_{refm}^{1/2}\log\left(C_{refm}^{-1/2}C^{\left(n\right)}C_{refm}^{-1/2}\right)C_{refm}^{1/2}\right)$$

These $z_{m}^{\left(n\right)}$ are concatenated to formulate the feature vector $z^{\left(n\right)}$ with a dimensionality of m·d, where *d* represents the length of individual projection vectors. Subsequently, the composite set $\left\{z^{\left(n\right)},\;t^{\left(n\right)}\right\}$ is used for training a linear classifier.

This methodological framework augments the discriminative capacity by capturing the inherent Riemannian structure of EEG data across distinct tangent spaces.

### 3.2. Cholesky Decomposition Projections

Cholesky decomposition is a method of decomposing an SPD matrix into the product of a lower triangular matrix and its conjugate transpose. This technique is computationally efficient and stable, making it suitable for real-time applications.

The steps involved in the Cholesky decomposition approach are as follows:

– Computation of covariance matrices: Similar to the tangent space projection method, the covariance matrices of EEG signal trials are computed.
– Cholesky decomposition: Each covariance matrix $C^{\left(n\right)}$ is decomposed into a lower triangular matrix $L^{\left(n\right)}$ such that $C^{\left(n\right)}=L^{\left(n\right)}{\left(L^{\left(n\right)}\right)}^{⊤}$.
– Feature extraction: The elements of the lower triangular matrix $L^{\left(n\right)}$ are used as features for classification. These elements capture the essential characteristics of the covariance matrix in a reduced form.
– Application of classifiers: The extracted features are used as inputs for classifiers such as the GG-FWC, which constructs the output through a functionally weighted linear combination of the input variables.

### 3.3. The Monolithic GG-FWC

**Figure 1:**
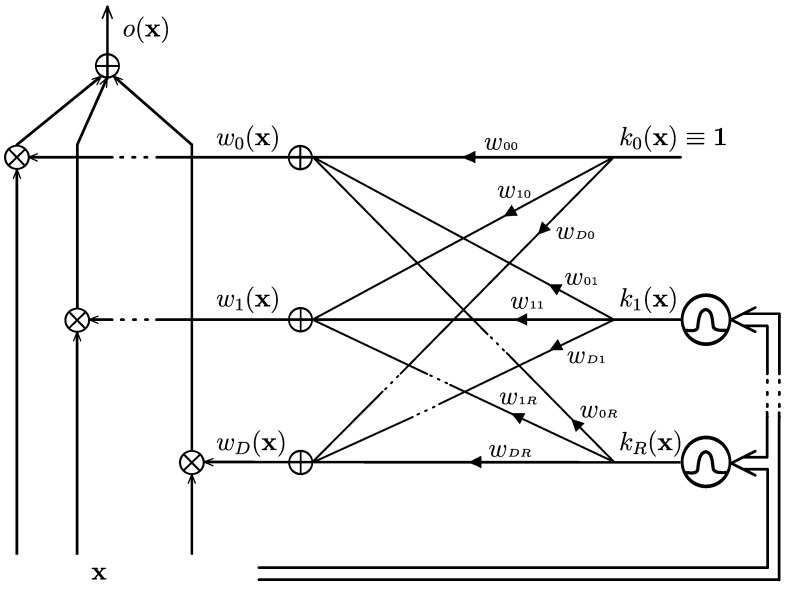
Gate generated functional weight classifier [22], where x: input sample; *o*: output; $k_{r}\left(x\right)$: *r*-th kernel output; $w_{d}\left(x\right)$: *d*-feature weight.

The architecture of the monolithic GG-FWC presented in Figure 1 was first introduced by [22]. The paper outlines the derivation of this architecture from a mixture of experts in an ensemble with linear learners. This process involves reordering summations in the output formula and selecting a kernel gate, which imparts sufficient expressive power to the resulting machine. Importantly, a complex training process is unnecessary if kernels are chosen beforehand using a suitable algorithm.

The form of the GG-FWC output is:

(3) $$o\left(x\right)=\sum_{d=0}^{D}\left(\sum_{r=1}^{R}w_{dr}k_{r}\left(x\right)\right)x_{d}$$

where $x_{0}=1$, $k_{0}\left(x\right)=1$, $x={\left[x_{1},\ldots ,x_{D}\right]}^{⊤}$ is a *D*-dimensional observation, $k_{r}\left(x\right)$, r=1,…,R are kernels’ outputs, and $w_{d,r}$ is the weight of kernel *r* and radial basis function (RBF) that compute the weight of feature *d*. With the change of variables $z_{s}\left(x\right)=k_{r}\left(x\right)x_{d}$, where s=r(d+1), Equation (3) becomes:

(4) $$o\left(x\right)=\sum_{s=0}^{S-1}w_{s}z_{s}\left(x\right)$$

This equation represents a linear model of the transformed variable set $\left\{z_{s}\left(x\right)\right\};\;s=1,\ldots ,R\left(D+1\right)$. Hence, the parameters can be determined using a maximal margin (MM)/linear SVM algorithm [23,24,25].

The GG-FWC can be viewed as a fusion of a linear classifier (the $\left(\left\{x_{d}\right\}\right)$ elements) and a nonlinear classifier (the rest of the elements). This could be beneficial, as [26] showed successful results when combining double projection with a linear classifier.

### 3.4. Proposed Method

**Figure 2:**
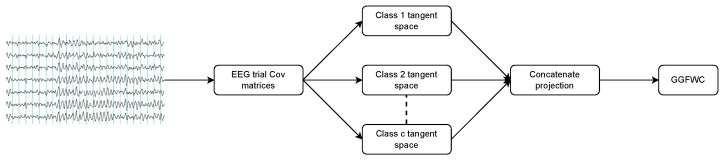
M-TSP followed by GG-FWC classifier.

In this study, we propose two distinct methodologies for using raw EEG covariance matrices in MI-BCI tasks. These methods diverge in their strategies for handling covariance matrices. The first one, illustrated in Figure 2, is inspired by our previous work [8]. It involves calculating the representative mean of each class sample, followed by mapping all samples into the tangent spaces corresponding to the computed class means. The resulting projections are concatenated and are used as input for the GG-FWC classifier. Algorithm 1 outlines the training procedure using M-TSP, whereas the prediction procedure is detailed in Algorithm 2.
**Algorithm 1** Training the M-TSC model**Require:** $\left(C^{n},t^{n}\right)$ the set of training data n=1…N **for** m=1 to *M* **do**  $C_{refm}=$ the Riemannian mean of class *m* **end for** Z= empty-matrix() **for** n=1 to *N* **do**  **for** m=1 to *M* **do**   $z_{m}^{\left(n\right)}=upper\left(C_{refm}^{1/2}\log\left(C_{refm}^{-1/2}C^{\left(n\right)}C_{refm}^{-1/2}\right)C_{refm}^{1/2}\right)$  **end for**  Z[n,] = concatenate$\left(z_{0}^{\left(n\right)},z_{1}^{\left(n\right)},\ldots ,z_{M}^{\left(n\right)}\right)$ **end for** model = GGFWC(model parameters) model = model.fit(Z, t)             ▹t the vector of all $t^{n}$ Return (model)


**Algorithm 2** Class Prediction with M-TSC**Require:** model: trained model **Require:** $C^{n}$: new sample covariance matrix **Require:** $C_{refm}$: the Riemannian mean of class *m*  **for** m=1 to *M* **do**     $z_{m}^{\left(n\right)}=upper\left(C_{refm}^{1/2}\log\left(C_{refm}^{-1/2}C^{\left(n\right)}C_{refm}^{-1/2}\right)C_{refm}^{1/2}\right)$  **end for**  z = concatenate$\left(z_{0}^{\left(n\right)},z_{1}^{\left(n\right)},\ldots ,z_{M}^{\left(n\right)}\right)$  *y* = model.predict(z)  Return (y)


**Figure 3:**
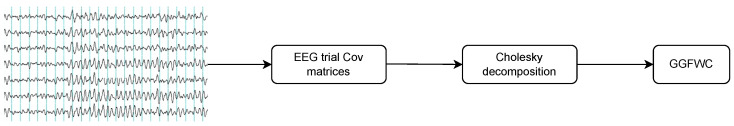
Cholesky decomposition followed by GG-FWC classifier.

In contrast, as shown in Figure 3, the second method employs Cholesky decomposition as a descriptor for the covariance matrices, and the resulting features are used as input for the GG-FWC classifier. The Cholesky training procedure is described by Algorithm 3, while the prediction procedure is presented by Algorithm 4.
**Algorithm 3** Training the Cholesky model**Require:** $\left(C^{n},t^{n}\right)$ the set of training data n=1…N Z= empty-matrix() **for** n=1 to *N* **do**  Z[n,] = cholesky_decomposition($C^{n}$) **end for** model = GGFWC(model parameters) model = model.fit(Z, t)             ▹t the vector of all $t^{n}$ Return (model)


**Algorithm 4** Class prediction with Cholesky model**Require:** model: trained model **Require:** $C^{n}$: new sample covariance matrix  z = cholesky_decomposition($C^{n}$)  *y* = model.predict(z)  Return (y)


To apply the GG-FWC model, it is necessary to determine the *R* RBF centroids and dispersions. In this work, we have chosen one of the design procedures presented in [22] for this purpose. Specifically, centroids are determined using the *k*-means algorithm, while dispersion is calculated as a sample estimate of the variance of the samples associated with each *k*-means centroid. To find the most appropriate dispersion value, a “scale” factor δ is applied.

For the proposed methodologies, both approaches share the same three hyperparameters associated with the GG-FWC model. These hyperparameters are:

- *R*: the number of RBF units;
- *C*: the parameter for the MM algorithm;
- δ: the scale factor for the dispersion of RBF.

The hyperparameter determination process is carried out via Bayesian optimization by leveraging the scikit-optimize package [27]. This approach entails exploring diverse parameter ranges, encompassing:

- Variation in the number of centroids, spanning from 2 to 30;
- δ, ranging from 1 to 400;
- Parameter *C*, encompassing a specified range of values: [0.0001, 0.001, 0.01, 0.1, 1, 10, 100, 1000].

Moreover, this optimization procedure involves executing a total of 300 runs to thoroughly assess the parameter space.

## 4. Experiment

To showcase the robustness and efficacy of the proposed method, this section conducts a series of experiments utilizing the two datasets introduced earlier. The acquired results of these experiments are meticulously compared with those obtained from all variants of multiple tangent space classification (M-TSC) previously introduced in our prior work [8]:

- LR M-TSC1: This iteration involves the multiple tangent space projection of covariance matrices, succeeded by the concatenation of the resultant data, followed by classification using LR.
- SVM M-TSC1: In this variation, the multiple tangent space projection is applied, and the ensuing data are concatenated and classified using SVMs.
- LR M-TSC2: In this version, the approach involves the multiple tangent space projection of covariance matrices. The resultant data from diverse projections undergo concatenation and subsequent normalization to achieve zero means and unity standard deviation before being employed as input for a logistic regression classifier.
- SVM M-TSC2: After applying the multiple tangent space projection of covariance matrices in this variant, the resulting data from various projections are concatenated and normalized to zero means and unity standard deviation prior to being utilized as input for a SVM.

By subjecting our model to individual comparisons with each version, we acquire a detailed perspective, facilitating a meticulous analysis of how this method evolves in its performance across distinct users. This approach offers comprehensive insights into the customized efficacy and adaptability of the proposed method for diverse user profiles.

### 4.1. Dataset

The objective of this study is to evaluate the performance of the proposed model and enable a comparative analysis with the outcomes of our preceding research [8]. To achieve this, we employ the same pair of public datasets utilized in the initial paper, denoted as Dataset B and Dataset A, both originating from the BCI Competition of 2008. For the remainder of this paper, we will refer to these datasets as DS1 and DS2.

- DS1: This dataset [28] comprises raw labeled EEG data obtained from nine subjects and recorded through three channels (C3, C4, and Cz). Data acquisition was based on the mental reproduction of two different gestures: one corresponding to the left hand (class 1) and the other to the right hand (class 2). Each subject completed a total of five sessions, which consisted of 120 trials per session. The dataset is divided into three sessions for training and two sessions for testing, providing 360 trials for training and 240 trials for testing for each subject. In this dataset, the subject’s gender is not known.
- DS2: This dataset [29] includes raw labeled EEG data collected through a 22-electrode helmet from a cohort of nine subjects: four females and five men. Each participant actively attended two separate data capture sessions, during which they performed mental simulations that covered a variety of gestures related to left hand (class 1), right hand (class 2), both feet (class 3), and tongue (class 4) movement. For the purpose of performance comparison with the preceding work, only data corresponding to two classes, the right-hand and left-hand, were selectively retained. The dataset is divided into one session for training and one session for testing, with each session consisting of 120 trials per subject.

### 4.2. DS1 and DS2 Analyses

This section delineates the findings derived from experiments conducted using datasets 2a and 2b of the BCI Competition IV. A comparative analysis will be undertaken, examining the obtained results with different versions of M-TSC model proposed in [8]. As the DS2 dataset includes gender information, a gender-based analysis will be conducted to discern the potential impact of gender on the observed results. This comprehensive evaluation aims to provide deeper insights into the performance of the proposed method across diverse datasets, considering gender as a potential influencing factor.

The following figures provide a comprehensive overview of the results derived from the experiments conducted with datasets DS1 and DS2. This includes a comparative analysis in terms of accuracy against the performance benchmarks set by M-TSC.

#### 4.2.1. DS2 Dataset: Key Findings and Outcomes

**Figure 4:**
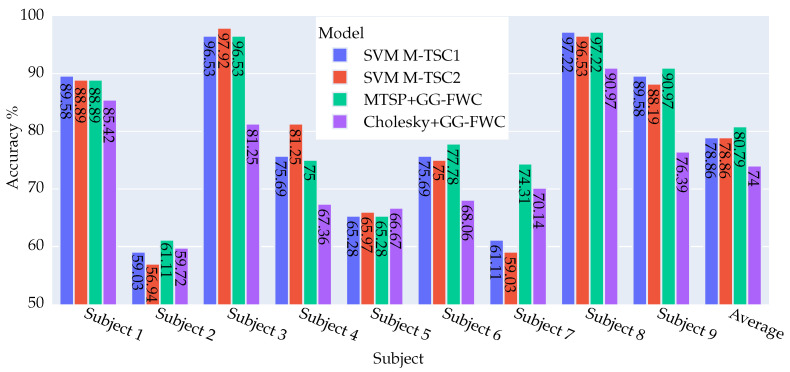
Visual representation of accuracy results for four approaches—SVMM-TSC1, SVMM-TSC2 [8], M-TSP + GG-FWC, and Cholesky + GG-FWC—for nine subjects and their averages using dataset DS2. M-TSP + GG-FWC showed the best performance.

In the results outlined in Figure 4, a meticulous examination of the new method against (SVM M-TSC1) reveals a notable enhancement across four subjects (2, 6, 7, and 9). User 7 particularly shines, with a remarkable improvement of 13.2%, while two subjects exhibit a marginal decline of −0.69%. It is important to highlight that the accuracy rates for the remaining subjects remain consistent.

When comparing with (SVM M-TSC2), there is a discernible uptick in accuracy for five subjects (2, 6, 7, 8, 9). Notably, users 9 and 2 demonstrate standout performances, with improvements of 15.28% and 4.17%, respectively, while three subjects (3, 4, and 5) register a reduction, with subject 4 showcasing a substantial deterioration of −6.25%.

**Figure 5:**
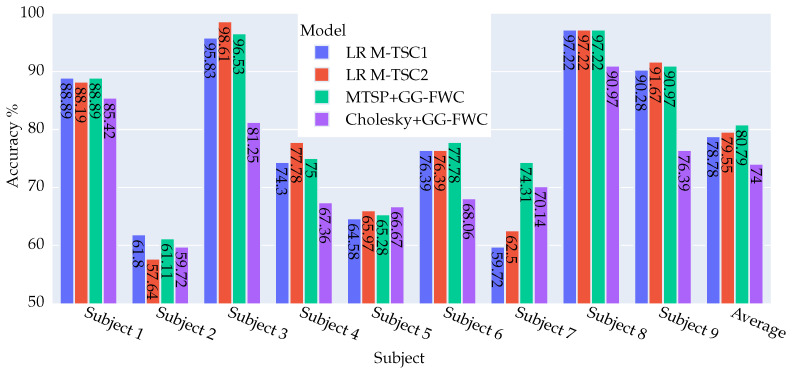
Visual Representation of accuracy results for four approaches—LR M-TSC1, LR M-TSC2 [8], M-TSP + GG-FWC, and Cholesky + GG-FWC—for nine subjects and their averages using dataset DS2. M-TSP + GG-FWC showed the best performance.

Figure 5 undertakes a comparative analysis between the current results and those of (LR M-TSC1). In this context, nearly all users exhibit improvement or maintain their previous standings, except for user 2, who registers a slight decline of −0.69%. Notably, user 7 demonstrates a significant improvement of 14.59%, which is particularly noteworthy.

In comparison with (LR M-TSC2), four instances of deterioration (subjects 3, 4, 5, 9) are evident, with two being particularly significant at 2.08% and 2.78%. On a positive note, three cases demonstrate improvement, with subjects 2 and 7 showing substantial gains of 3.47% and 11.81%, respectively. These nuanced insights further enrich the understanding of the method’s performance across diverse subjects and comparative models.

In the two figures, a comparative analysis of the Cholesky method versus the different versions of M-TSC underscores the consistent inferiority of the latter for the majority of subjects. In particular, subjects 3 and 9 stand out, displaying a substantial deterioration that exceeds 11%. On the other hand, subject 7 shows a significant improvement ranging between 7.64% and 11.11% for LR M-TSC2 and SVM M-TSC2, respectively, relative to the Cholesky method.

Upon comparing M-TSP + GG-FWC with Cholesky + GG-FWC, a notable disparity in performance emerges, with M-TSP demonstrating a significant superiority with an average margin of 6.79% over Cholesky. Remarkably, M-TSP outperforms the Cholesky method across all subjects except for subject 5, where the Cholesky method exhibits a slight advantage of 1.39%. The most notable performance differentials are observed in subjects 3 and 9, where M-TSP outperforms the Cholesky method by 15.28% and 14.58%, respectively.

#### 4.2.2. DS1 Dataset: Key Findings and Outcomes

**Figure 6:**
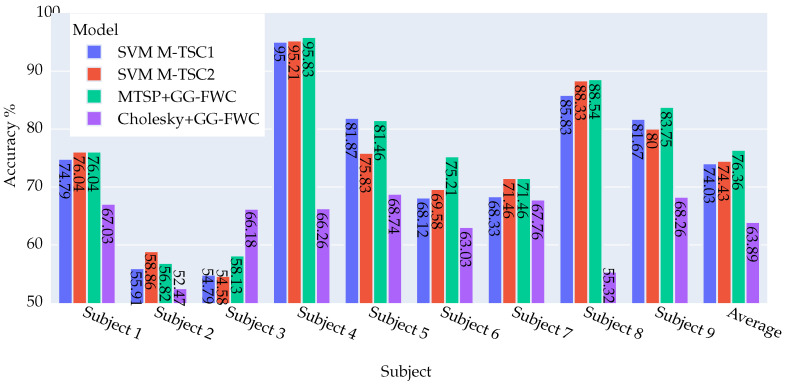
Visual representation of accuracy results for four approaches—SVM M-TSC1, SVM M-TSC2 [8], M-TSP + GG-FWC, and Cholesky + GG-FWC—for nine subjects and their averages using dataset DS1. M-TSP + GG-FWC showed the best performance.

In Figure 6, we conduct a comprehensive comparison between the proposed method and the SVM M-TSC1 model. The results indicate remarkable superiority of the new method among almost all users, with particularly significant improvements exceeding 2% observed for subjects 3, 7, 8, and 9. It is important to highlight a marginal deterioration in performance of −0.41% for user 5.

The comparison with SVM M-TSC2 reveals a substantial enhancement of 5.63% for users 5 and 6 followed by 3.75% and 3.55%, respectively, for users 9 and 3. However, user 2 demonstrated a decrease in performance of 2.04%.

**Figure 7:**
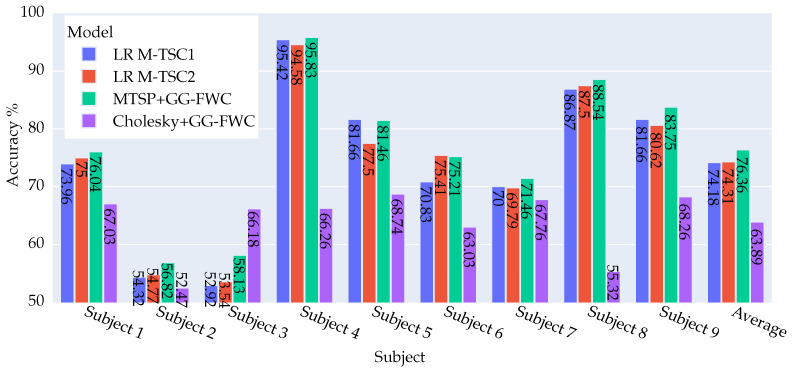
Visual representation of accuracy results for four approaches—LR M-TSC1, LR M-TSC2 [8], M-TSP + GG-FWC, and Cholesky + GG-FWC—for nine subjects and their averages using dataset DS1. M-TSP + GG-FWC showed the best performance.

Within Figure 7, the outcomes obtained using the proposed model in this article are compared with those obtained using the LR M-TSC1 approach. A compelling observation is that, in this case, all results either match or surpass those of the prior study. In particular, user 3 showed a substantial improvement of 4.37%. However, user 2 demonstrated a minor decrement of −0.2%, which is considered statistically insignificant.

While comparing with LR M-TSC2, a notable improvement exceeding 3% is observed for users 3, 5, and 9. We highlight the accuracy associated with subject 3, which showcased a notable improvement of 4.59%. Similarly, as in the previous case, a negligible deterioration of −0.2% was observed in the case of subject 6.

The comparison across both charts reveals a notable inferiority in the performance of the Cholesky method compared to the different versions of M-TSC, with an average deterioration of approximately 10%. Particularly notable is the significant deterioration observed for subjects 4 and 8, where the performance gap exceeds 28%. However, subject 3 presents a contrasting scenario, with the Cholesky method exhibiting a superiority of over 11%.

When comparing M-TSP + GG-FWC, it becomes clear that this method generally outperforms Cholesky + GG-FWC for eight out of nine subjects. Notably, M-TSP demonstrates exceptional performance for subjects 4 and 8, showcasing a superiority over the Cholesky method of 29.57% and 33.22%, respectively. In contrast, the Cholesky method exhibits better performance than M-TSP for subject 3, showing an improvement of 8.05%.

These findings accentuate the robust performance of the M-TSP + GG-FWC model and showcase its consistency and notable enhancements across various scenarios.

#### 4.2.3. Summary of Model Achievements

The comprehensive analysis performed highlights a compelling advantage of the proposed model. With regard to dataset DS1, a remarkable mean accuracy of 76.36% was achieved, demonstrating consistent improvements of 2.33%, 1.93%, 2.18%, and 2.05% compared to SVM M-TSC1, SVM M-TSC2, LR M-TSC1, and LR M-TSC2, respectively. Focusing on the DS2 dataset, our model demonstrated even greater efficiency, with a mean accuracy of 80.79%. This represents a substantial improvement of 1.93% compared to the two M-TSC variants using SVM as a classifier. Furthermore, our model outperformed LR M-TSC1 and LR M-TSC2 with notable improvements of 2.01% and 1.24%, respectively. These results highlight the robust performance of the proposed model on various datasets.

On the other hand, the comparison between M-TSP and the Cholesky method reveals stronger predictive performance for M-TSP across most subjects. However, there are instances where the Cholesky method exhibits competitive accuracy scores. These findings underscore the importance of evaluating model performance on a large dataset to obtain a comprehensive understanding of its effectiveness and suitability for different applications.

### 4.3. Gender-Based Analysis

A comparison based on gender was conducted using the gender information provided in the DS2 dataset. The dataset included four female subjects (1, 2, 4, and 6) and five male subjects (3, 5, 7, 8, and 9). This comparative analysis aims to investigate the impact of gender on the results of an MI-BCI system. Additionally, this exploration provides an opportunity to evaluate the proposed model in the context of gender dynamics, offering information on potential variations or influences that may be present. This gender-centric assessment contributes to a more nuanced understanding of the performance and applicability of the BCI model, enriching the overall analysis of motor imagery outcomes.

**Figure 8:**
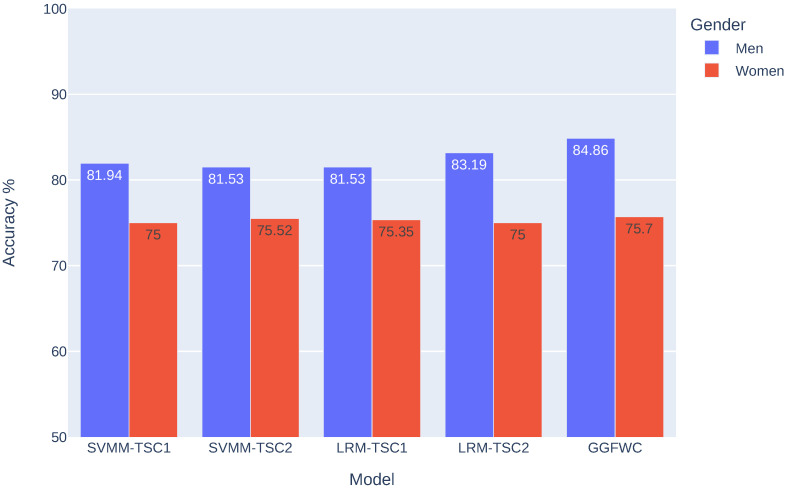
Exploring gender-based differences in model accuracy: comparative evaluation of five models, including the four versions of M-TSC [8] and GG-FWC.

The visual representation in Figure 8 offers a comprehensive perspective on gender-specific mean values across various versions of the M-TSC model and the model proposed in this paper. Significantly, in all scenarios, the mean accuracies for men consistently surpass those for women, with margins of 6.94%, 6.01%, 6.18%, and 8.19% for SVM M-TSC1, SVM M-TSC2, LR M-TSC1, and LR M-TSC2, respectively. It is interesting to highlight that the introduction of the GG-FWC model widens the gender gap to 9.16%, underscoring a performance advantage for men. Through an in-depth comparison between GG-FWC and M-TSC, the superiority of the proposed model becomes apparent across all four versions of M-TSC, demonstrating remarkable consistency in performance. Examining gender-specific outcomes, women experience modest improvements ranging from 0.17% to 0.7%, with SVM M-TSC2 exhibiting the smallest enhancement and SVM M-TSC1 showcasing the most substantial increase. In contrast, the male group experiences notably significant improvements. The slightest enhancements are noted in comparison to LR M-TSC2 at 1.67%, followed by a 2.92% improvement relative to SVM M-TSC1, and finally, the most significant advancement is 3.33%. The findings revealed a consistent and notable advantage for men across all cases, evident not only in terms of the accuracy rate but also in the improvement rate. This gender disparity in performance merits further exploration and consideration for understanding the factors of the model’s effectiveness in different demographic contexts.

## 5. Discussion and Future Directions

This study introduces two methodologies aimed at improving the recognition of EEG brain signals associated with motor imagery, focusing on covariance matrices. The first approach involves employing a multiple tangent space projection technique for covariance matrices, while the second method utilizes Cholesky decomposition. The resulting sets of data obtained from both methodologies were utilized separately as inputs for the GG-FWC classifier. The effectiveness of the model was evaluated using two distinct datasets to assess its classification accuracy. Initially, the performance of M-TSP combined with GG-FWC was compared with the M-TSP approach, which uses traditional classifiers. Then, a comparative analysis was performed between M-TSP and Cholesky decomposition.

The analysis demonstrates considerable enhancements for M-TSP + GG-FWC across both datasets, exceeding 11% improvement in DS2 and 5% in DS1 for some subjects. Furthermore, when comparing M-TSP and the Cholesky method, it becomes apparent that M-TSP generally outperforms the Cholesky method, despite instances where the Cholesky method showed better results for certain subjects.

Due to the lack of additional information, we are unable to provide further explanation for these differences in improvements. Notably, not all users possess the capability to adequately modulate their brain activity for effective control of an MI-BCI. Factors such as demographic and psychological measures play a crucial role in influencing individual abilities. The absence of this essential information in the used databases constrains the depth of user-specific studies. Consequently, we intend to create our own database to conduct a more exhaustive and in-depth study, with the ultimate goal of developing a real-time application for deployment on robots.

On the other hand, a gender-based analysis was conducted, revealing an advantage for men. In contrast to our findings, certain studies, including those referenced in [30,31], have indicated a notable advantage for women over men. This disparity motivates us to shift our focus towards a meticulous gender-based analysis in future investigations. In our future work, we will conduct this analysis using more extensive datasets that cover a larger number of subjects to provide a comprehensive exploration of gender-related nuances.

These findings underscore the potential of M-TSP to significantly enhance the performance of motor imagery-based BCI systems. The demonstrated improvements suggest that M-TSP could be effectively integrated into practical BCI applications, potentially improving user experiences and the precision of control in various real-world scenarios. This is particularly relevant for developing assistive technologies for individuals with severe motor impairments, where improved accuracy directly translates to better usability and functionality of BCI systems.

The findings of this paper have several important implications:

– Enhanced BCI performance: The proposed methodologies significantly improve the accuracy of motor imagery classification, which can lead to more effective and reliable BCI systems for neurorehabilitation and assistive technologies.
– Personalized BCI systems: The gender-based analysis reveals performance differences between male and female subjects, highlighting the need for personalized BCI systems that can adapt to individual user characteristics.
– Robust feature extraction: The use of covariance matrices and the integration of linear and nonlinear features through the GG-FWC classifier demonstrate the potential for robust feature extraction methods that can capture the complex dynamics of EEG signals.

Based on the findings and limitations of this study, several directions for future research are suggested:

– Development of a comprehensive database: Create a comprehensive database that includes demographic and psychological measures to enable more detailed user-specific studies. This will help with understanding the factors influencing individual abilities to control MI-BCIs.
– Real-time application and deployment: Develop and test a real-time BCI application for deployment on robotic systems such as prosthetics, exoskeletons, and robotic arms in order to validate the practical applicability of the proposed methodologies.
– Exploration of additional features: Investigate the incorporation of additional features such as EEG signal complexity measurements and feature fusion techniques to further enhance the performance and robustness of MI-BCI systems.
– Gender-based adaptations: Conduct more extensive gender-based analyses using larger datasets to explore and develop gender-specific adaptations in BCI systems, ensuring optimal performance for both male and female users.
– Integration with deep learning: Explore the integration of the proposed methodologies with deep learning techniques such as convolutional neural networks and recurrent neural networks to leverage their powerful feature learning capabilities.

## 6. Conclusions

This study introduces innovative methodologies for enhancing EEG signal classification in MI-BCIs. Using M-TSP and Cholesky decomposition for covariance matrices, along with the GG-FWC, we demonstrate significant improvements in classification accuracy. Our comparative analysis with M-TSC benchmarks using datasets DS1 and DS2 from the BCI Competition IV shows that our methods consistently outperform traditional approaches. The M-TSP method, in particular, shows superior performance, highlighting its potential for practical BCI applications. The gender-based analysis underscores the need for personalized BCI systems that take into consideration performance differences between male and female subjects. These advancements contribute to the development of more robust, accurate, and efficient MI-BCI systems, paving the way for future research and applications in neurorehabilitation and assistive technology.

## Data Availability

The original data presented in the study are included in the article.
